# Experimental Measurement of the Thermal Conductivity of Fused Deposition Modeling Materials with a DTC-25 Conductivity Meter

**DOI:** 10.3390/ma16237384

**Published:** 2023-11-27

**Authors:** Antonio Rodriguez, Juan Pablo Fuertes, Añaterve Oval, Gurutze Perez-Artieda

**Affiliations:** 1 Engineering Department, Campus de Arrosadía S/N, Public University of Navarre (UPNA), 31006 Pamplona, Spain; antonio.rodriguez@unavarra.es (A.R.); aoval@arquimea.com (A.O.); gurutze.perez@unavarra.es (G.P.-A.); 2 Arquimea Research Center, Camino de Las Mantecas S/N, Parque Urbano de Las Mantecas, Edificio NANOtec, 38320 San Cristóbal de La Laguna, Spain

**Keywords:** additive manufacturing, fused deposition modeling, filament, thermal conductivity, 3D printing, DTC-25

## Abstract

The expansion and low cost of additive manufacturing technologies have led to a revolution in the development of materials used by these technologies. There are several varieties of materials that can be used in additive manufacturing by fused deposition modeling (FDM). However, some of the properties of these materials are unknown or confusing. This article addresses the need to know the thermal conductivity in different filaments that this FDM technology uses, because there are multiple applications for these additive manufacturing products in the field of thermal insulation. For the study of thermal conductivity, the DTC-25 commercial conductivity measurement bench was used, where the tests were carried out on a set of seven different materials with 100% fabrication density—from base materials such as acrylonitrile butadiene styrene (ABS) or polylactic acid (PLA), to materials with high mechanical and thermal resistance such as thermoplastic polyurethane (TPU), polyether ether ketone (PEEK), and high-performance polyetherimide thermoplastic (ULTEM), to materials with metal inclusions (aluminum 6061) that would later be subjected to thermal after-treatments. This study shows how the parts manufactured with aluminum inclusions have a higher thermal conductivity, at 0.40 ± 0.05 W/m·K, compared to other materials with high mechanical and thermal resistance, such as TPU, with a conductivity of 0.26 ± 0.05 W/m·K.

## 1. Introduction

The great advance of 3D printing systems using fused deposition modeling (FDM) technology has allowed their use in a variety of areas thanks to their ability to produce parts of great geometric complexity in a relatively fast, easy, and economical way, reducing the number of processing stages. In addition, FDM allows for the use of different thermoplastic materials, such as polylactic acid (PLA), acrylonitrile butadiene styrene (ABS), or polyether ether ketone (PEEK), with minimal changes in the components and configuration.

To allow the use of this technology in certain applications with thermal requirements, a critical property is the thermal conductivity of the model, which may vary depending on the amount of air inside the part [1], the material used [1,2,3,4], the direction of heat flow with respect to the printing direction [5,6,7], and sintering or post-treatment in filaments including metallic particles [8].

The addition of copper particles can significantly increase thermal conductivity [2], especially with contents greater than 20% by mass, going from a conductivity of 0.12 W m^−1^ K^−1^ with a pure PLA sample to 0.35 W m^−1^ K^−1^ with a copper particle content of 40% by mass. In addition, it has been verified that adding 20% Polymethyl methacrylate (PMMA) particles by mass enhances this increase in conductivity, increasing by 43% with respect to the same material without PMMA, and reaching a conductivity of 0.49 W m^−1^ K^−1^.

Laureto et al. [1], in turn, analyzed the effects of the addition of particles of different metals and compared them with the Lichtenecker equation, which predicts the conductivity of the material as a function of the conductivity of the polymer matrix and the concentration in volume and conductivity of the metal particles. Likewise, it studies the effect of the porosity of the material, concluding that it is necessary to minimize the air content and maximize the amount of metal particles to increase thermal conductivity.

In addition, referring to additive manufacturing using fused deposition technology, articles can be found in which the inclusion of carbon fibers is used to improve thermal conductivity. Ibrahim et al. [4] analyzed the variations in the conductivity of a nylon matrix sample, with different layer configurations and fiber directions, obtaining the maximum conductivity with fibers in the direction of heat flow and reaching a conductivity 11 times higher than that of the base material. Likewise, lattice structures have been proven to be one of the best choices ever since their inception, for various structural and other commercial applications, due to their enhanced mechanical properties, especially in the case of vibration isolation, where band gaps play a vital role [9].

In the case of post-processing like the sintering of materials with copper particles, the thermal conductivity can be strongly increased, as noted by Ebrahimi et al. [8]. Starting with a material whose average volume content is 39.3% and whose conductivity is 1.5 W/K·m, it increases the copper concentration to 42.3% and reduces the porosity by sintering, resulting in a conductivity of 25.5 W/K·m.

Numerous studies have identified considerable challenges in the properties of FDM-printed components that cannot be addressed solely through optimal printing conditions [10]. On the one hand, residual stresses caused by non-uniform heating and cooling cycles during printing have been detected, which are connected to issues with surface roughness, mechanical strength, and dimensional accuracy. These cycles produce uneven temperature gradients, leading to deformations and defects, including shrinkage, warping, and twisting [11]. Another challenge is the presence of interlayer voids, which weaken the parts and contribute to mechanical failures. Partial neck growth voids are particularly significant in causing voids in FDM, resulting from incomplete neck growth between adjacent chords during the sintering process [12]. Problems that arise with solidification prior to full coalescence can stem from inherent features such as incomplete filling and incoherent material flow [13]. Additionally, addressing the staircase effect phenomenon that leads to surface roughness presents another challenge [10]. Various post-processing strategies, including mechanical and chemical techniques, have been studied to optimize the surface finish of FDM-manufactured parts [14,15,16]. The ultimate challenge lies in achieving automation of these processes for efficient large-scale production [17,18].

In this work, the thermal conductivity of AA6061 and several polymers, including ABS, TPU, PLA, PEEK, and PEI (ULTEM 1010 and ULTEM 9085), was studied. Acrylonitrile butadiene styrene (ABS) was among the earliest materials used in 3D printing due to its chemical and abrasion resistance, along with its remarkable impact resistance. The material’s affordability further cements its place as one of the most sought-after 3D printing materials. It is crucial to consider that ABS plastic boasts a low melting point, meaning that it is unsuitable for extreme-temperature applications [18]. TPU filament is resistant to abrasion, oil, chemicals, and wear. TPU-printed parts display similar resistance to low temperatures, making them less prone to becoming brittle and challenging to handle. This flexible filament features excellent adhesion between layers and does not curl or delaminate while being 3D printed. Additionally, it is capable of withstanding significantly higher compressive and tensile forces than other more common materials, such as PLA and ABS [19].

Since 1990, polylactic acid (PLA) has been marketed as a biodegradable polymer. It is extensively researched and widely used due to its versatility in the market. PLA is also known for its easiness to print with compared to other plastics (210 °C). Additionally, it is an FDA-approved material with applications in the food and biomedicine industries [18].

Polyetheretherketone (PEEK) and polyetherimide (PEI) are examples of special engineering plastics with exceptional mechanical properties and high heat resistance [20]. PEEK’s biocompatibility and superior mechanical characteristics make it a potential biomaterial that can substitute metallic or ceramic components in fields such as biomedicine or aerospace, thereby opening up interesting prospects [21]. The use of FDM with PEEK is challenging due to its high melting temperature and viscosity. These obstacles must be overcome to fully achieve the potential of PEEK in advanced engineering applications. The PEI material (ULTEM 1010 and ULTEM 9085) exhibits exceptional thermal resistance when compared to other studied materials. It is able to maintain a constant maximum working temperature of around 200 °C while displaying minimal variation in its mechanical properties. These properties make it an ideal candidate for use in creating molds—such as those subjected to high pressure and Autoclave temperature values—including short-cycle injection molding tools and carbon fiber laminating tools. In all these applications, an understanding of thermal conductivity is a crucial factor in producing molds that are faster, simpler, and less expensive than current steel or aluminum molds [22].

In this context, different base polymer materials have been studied to compare their thermal conductivities and understand how 3D printing can influence their thermal capability.

An innovative filament comprising over 65% metal and the remainder PLA has been studied alongside various other base polymer materials. For thermal conductivity measurements, a commercial bench used for testing of metal components was adapted to enable high-confidence measurements on polymeric material.

## 2. Materials and Methods

### 2.1. Test Bench

**Figure 1:**
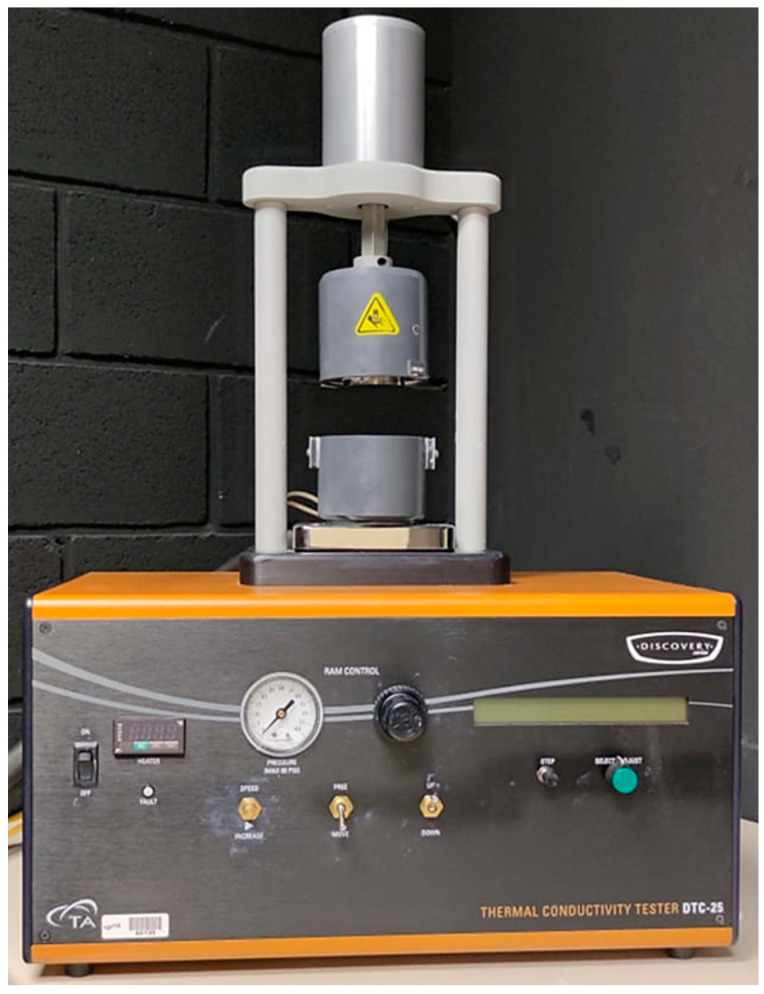
Test bench DTC-25 [24].

The test bench employed is a commercial system acquired by the Public University of Navarre that consists of a thermal conductivity meter, DTC-25, as shown in Figure 1. The DTC-25 thermal conductivity meter is a test instrument used for determination of the thermal conductivity of solid materials using the guarded heat flow method. Because of its simplicity to handle, small sample size, and short cycle time, it is ideally suited for quality control and the study of materials. Metals, ceramics, polymers, composites, glass, and rubber can all be tested accurately [23]. The main characteristics of the DTC-25 are shown in Table 1.

This instrument is factory-calibrated using specimens of known thermal resistance spanning the whole range of the instrument. Calibration reference sets are also available and an optional chiller to maintain fixed coolant temperature is recommended for optimal performance.

**Figure 2:**
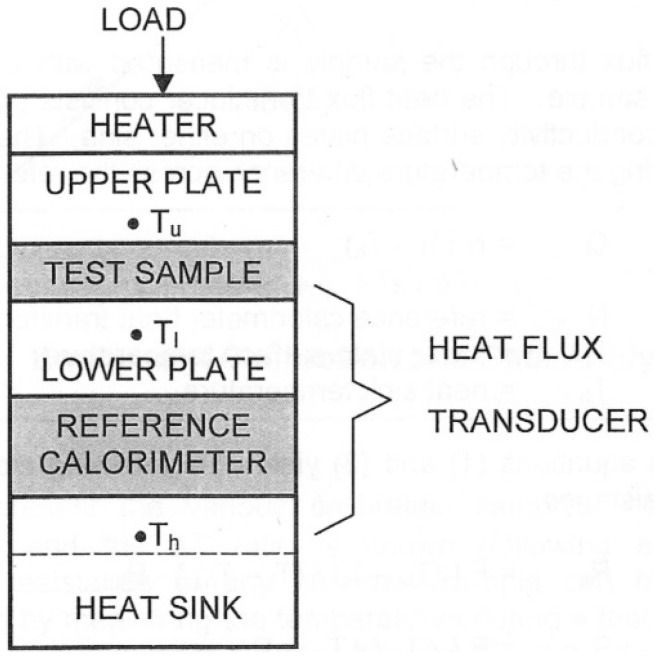
Guarded heat flow test method [25].

The measurement method, according to the ASTM E1530 specification [25], consists of placing the study sample under pressure between two polished metal surfaces. The upper one is heat controlled whilst the lower surface is part of a calibrated heat flux transducer and is connected to a liquid-cooled heat sink. As heat is transferred from the upper surface of the sample to its lower surface, a temperature gradient forms in the stack’s axial direction. A reproducible, pneumatic load is applied to the test stack to ensure a positive thermal contact. By measuring the temperature difference across the sample along with output from the heat flux transducer, it becomes possible to determine the thermal conductivity of the sample, given that its thickness is known, as shown in Figure 2.

To obtain values with less uncertainty in the DTC-25, the hot focus temperature source is set to 55 °C and the cold counterpart is set to 2 °C. The hot source is adjusted by means of an electrical resistance, while the cold temperature source is controlled by means of a thermostatic bath. Moreover, a pressurized nitrogen cylinder supplies the necessary pressure to move the actuator of the DTC-25 and exert pressure on the sample under study. A maximum pressure of 45 psi, 0.3 MPa, is exerted on the samples.

Once the test has been stabilized, the values shown on the bench display are processed by the manufacturer’s own software, showing the thermal conductivity value of the sample being tested.

### 2.2. Materials

The following subsection presents the materials used in this research work. The focus was set on affordable, common materials for 3D printing, including both polymers and metals. Specifically, we utilized aluminum 6061 as a metal and chose PLA, a biopolymer, and ULTEM, a polymer with superior mechanical properties, to be our polymeric materials. We conducted an analysis of thermal conductivity in different materials with varying properties to cover a wide range of applications. Table 2 shows the main characteristics and applications of the materials studied in this work.

### 2.3. Samples

As described in the previous subsection, seven different materials were studied in order to be classified according to the thermal conductivity, which was obtained from tests carried out in the DTC-25.

These are seven materials with distinct mechanical properties. To identify and exclude utility based on their thermal properties, it is necessary to determine their thermal conductivity. One of the limitations of this testing equipment is the pressure required to carry out the tests; on the DTC-25 test bench, it is 0.3 MPa. Therefore, we verified that all materials had higher maximum pressure limits; in the case of PEEK and PLA, the maximum pressure limit is 0.45 MPa, and it is up to 67 MPa in the case of thermoplastic polyurethane (TPU). It was also necessary to control the temperature of the materials. As shown in Table 3, the samples made with Filamet™ Aluminum 6061 and with a base material, PLA, had their maximum temperature limited to 55 °C. These specimens could not exceed the maximum temperature of the PLA, as the sample would lose its cohesion/integrity. In all the tests, we worked with temperatures below these limits.

#### Geometry

**Figure 3:**
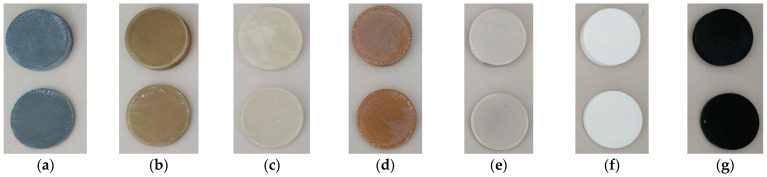
Study samples. FDM. From left to right: (**a**) aluminum, (**b**) PEEK, (**c**) TPU, (**d**) ULTEM 1010, (**e**) ULTEM 9085, (**f**) PLA 3080, (**g**) ABS.

Circular samples, shown in Figure 3, were manufactured using FDM printing with a standard diameter of 50 mm and two different thicknesses, a first thickness of 5 mm and a second of 10 mm. Both diameter and thickness were accurately measured, as can be seen in Table 4.

Different densities and fill patterns were produced using the different materials to achieve suitable mechanical [33] and thermal capabilities. However, finally, a density of 100% and rectilinear fill pattern with a 45° angle offset were selected as the lowest porosity and the best mechanical behavior. The remaining main parameters for printing were 0.8 nozzle diameter, 3.000 mm/min printing speed. 0.3 mm layer height, 1.75 mm filament diameter, and between 210 and 270 °C printing temperature depending on the material. In addition, the surface finish of the samples was established as between 1 and 10 microns depending on the material. This was necessary because together with the use of thermal contact paste, it allows the samples to be within the uncertainty of the bench, minimizing the influence of the thermal contact resistance between sample and equipment [34].

## 3. Results and Discussion

### 3.1. Hardness Tests

**Figure 4:**
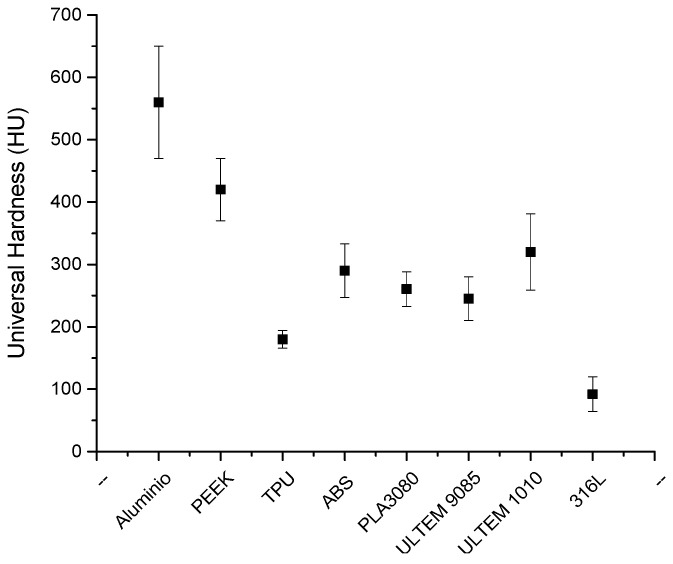
Hardness measurements of studied samples.

An approximation of the mechanical properties of the materials was arrived at through hardness tests [35]. A universal hardness test was used to compare all the samples. A maximum load of 1 N was applied. At least ten upload and download curves were studied for each material considering plastic and elastic deformation in the samples. Figure 4 shows an average of universal hardness at the maximum load.

The mechanical properties evaluated through universal hardness measurements showed values between 200 and 300 HU (Universal Hardness). Only PEEK was around 400 HU, and aluminum showed values up to 550 HU.

### 3.2. Data Acquisition

**Figure 5:**
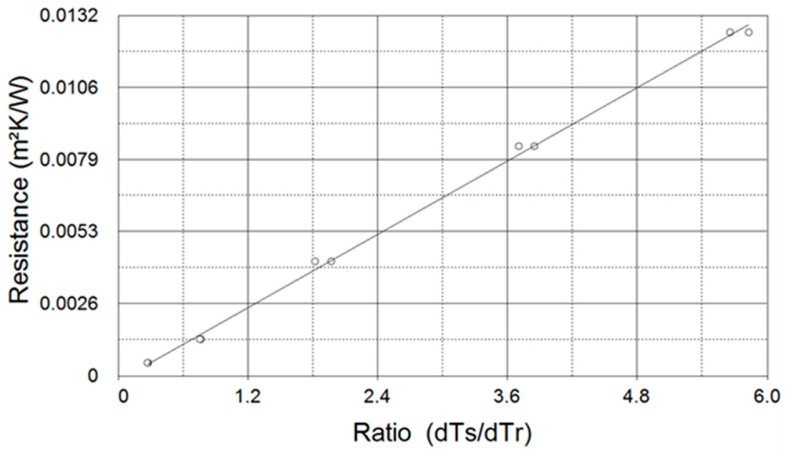
Calibration curve. DTC-25. Two measurements per standard.

To obtain accurate results, the system must be calibrated (Figure 5). To do this, the five standards provided by the manufacturer are tested, and the calibration curve is generated using the system software with the potential measurements taken for each standard after its study once stabilized. The temperature values measured with the respective probes are shown in voltage values. These values are the ones that will be entered into the system software for the calculation of the thermal conductivity of the sample.

In order to obtain more points on the calibration curve, two studies are carried out on each pattern. This way, different calibration measurements are obtained because, despite being the same piece under testing, the results may slightly differ, affected by the system itself, by the thermal paste used for the contact, or the environment (measurement deviation).

#### 3.2.1. Stability Criterion

The stabilization in the DTC-25 conductivity meter is carried out according to experience; it is related to the thickness of the study specimen, because for greater thicknesses, since they are materials of high thermal resistance, a longer study time will be required (Table 5).

#### 3.2.2. Uncertainty

The measurements were made within the limits set by the manufacturer, so that they lay within the reproducibility and uncertainty values provided. For this purpose, work was carried out within the ranges of thickness, size, and minimum conductivity of the sample indicated on the technical data sheet. DTC-25: 50 mm diameter, sample thickness greater than 0.1 mm, and theoretical conductivity between 0.1 and 20 W/m K.

To obtain an uncertainty value, we considered the randomness in the thermal conductivity measurements, performing N = 6 tests for each material studied. The mean value $\bar{k}$ of these samples was obtained by using Equation (1).

(1) $$\bar{k}=\frac{1}{N}\overset{N}{\underset{i=1}{\sum }}k_{i}$$

The standard uncertainty u(k) of the thermal conductivity k shown in Equation (2) was calculated in a similar way to the one shown in [35].

(2) $$u\left(k\right)=\sqrt{\frac{\sum_{i=1}^{N}{\left(k_{i}-\bar{k}\right)}^{2}}{N(N-1)}}$$

### 3.3. Thermal Conductivity Tests

**Figure 6:**
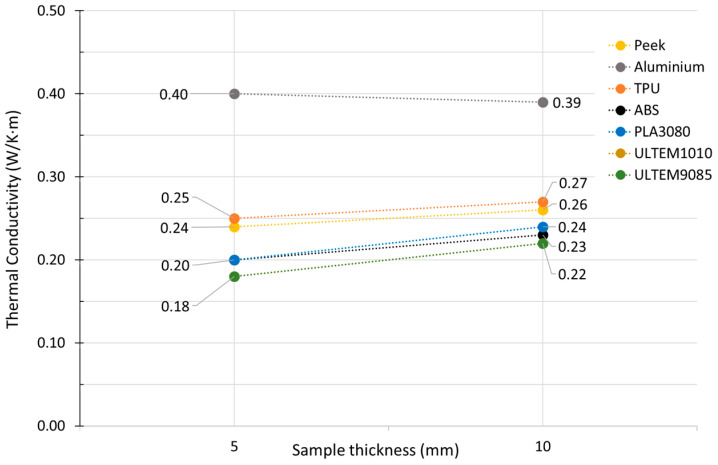
Average thermal conductivity.

The thermal conductivity values obtained for each thickness and material are shown in Figure 6, based on the results calculated with the DTC-25 bench software after entering the voltage readings (V) of each test.

In Table 6, we can see the results obtained for the average thermal conductivity with its measurement uncertainty. The material with the highest value is the Filamet™ of Aluminum 6061 of The Virtual Foundry (TVF), an innovative filament composed of more than 65% metal and the rest PLA, with a conductivity of 0.40 W/m·K. It is followed by the TPU, a thermoplastic polyurethane that combines hardness, elasticity, and mechanical resistance, so it maintains all the advantages of this elastomer, therefore being able to manufacture completely rigid parts, with a conductivity of 0.26 W/m·K.

This aluminum alloy, whose conductivity stands out among the rest of the materials used in FDM, arouses interest, above all, given what may happen with its conductivity after sintering (elimination of PLA from the alloy). The rest of the materials are within the thermal conductivity standard of thermoplastics, as shown in Table 6.

### 3.4. Discussion of Results

By comparing the results obtained in this study with the research conducted by M. C. Vu et al. [2], which examined the thermal conductivity of polylactic acid (PLA) composites with different copper (Cu) concentrations, we can highlight several findings regarding the impact of metal inclusions on thermal performance. The study by Vu et al. [2] demonstrated that for materials containing 100% PLA, the thermal conductivity was 0.12 W/m·K. As the mass percentage of Cu surpassed 20%, a notable enhancement in thermal conductivity to 0.35 W/m·K was achieved. This enhancement is ascribed to copper’s intrinsically high thermal conductivity. The outcomes of this research align with those of Vu et al. [2], wherein the same effect was observed when materials comprising more than 65% Al were examined in combination with PLA. A noteworthy advancement in thermal conductivity was achieved, exhibiting a value of 0.40 W/m·K. Notably, aluminum is acknowledged to have a slightly inferior thermal conductivity in comparison to copper despite being an excellent heat conductor. Hence, the observation that metallic composites with a considerable mass fraction of aluminum exceeded those with higher copper proportion accentuates the significance of optimizing the metallic inclusions’ mass percentages.

It is important to consider that thermal conductivity is influenced by factors beyond the presence of metallic inclusions, including the material’s porosity. This point was emphasized in the study by Laureto et al. [1], which highlights the importance of reducing air content and increasing metal particle content to enhance thermal conductivity. The significance of design and precise material control during the FDM process is further emphasized to prevent porosity from adversely impacting the final product’s heat transfer capabilities.

The comparison of Filamet™ Aluminum 6061 filament with other materials, including TPU, ABS, PEEK, PLA3080, ULTEM1010, and ULTEM9085, provides valuable insights into these materials’ thermal properties. Filamet™ Aluminum 6061 demonstrates remarkable thermal conductivity of 0.40 ± 0.05 W/m.K. This innovative filament, which comprises more than 65% metal and PLA, exhibits excellent potential for achieving superior thermal performance using high-metal materials, as previously mentioned.

Conversely, TPU, a thermoplastic polyurethane, exhibits an acceptable thermal conductivity of 0.26 ± 0.05 W/m·K. This thermoplastic material exemplifies its aptness in situations calling for a combination of mechanical strength and thermal properties due to its elasticity and mechanical robustness.

The thermal conductivities of the remaining materials, ABS, PEEK, PLA3080, ULTEM1010, and ULTEM9085, were all within the expected range for thermoplastics. These materials can now be used as a benchmark for the thermal behavior of FDM materials.

### 3.5. Results’ Limitations

The geometry chosen for this experiment relates to the limitations of the measuring equipment used. To compare the thermal conductivities of the various materials, the same conditions were maintained for all the samples in terms of geometry and test conditions. Given that changes in geometry can lead to very different outcomes. Future studies with other measuring equipment will be considered to further investigate this matter. To reduce porosity, a factor that affects the thermal conductivity of materials obtained through additive manufacturing, a rectilinear filling pattern with an angular displacement of 45° was selected. This same system was applied to all samples. Sintering or compaction could potentially enhance the porosity inherent to these processes, and a potential area for future research could be the application of post-additive manufacturing treatment techniques. Metal filaments or particles enhance thermal conductivity but also pose challenges for fabrication. While future research could investigate their potential for analysis, this study focuses on measuring commercially available and easy-to-fabricate materials.

This study’s findings are of great importance to research on additive manufacturing, particularly to that involved with FDM-based 3D printing. This research significantly contributes to our understanding of the impact of various factors such as metal inclusions and material choices on the thermal conductivity of FDM printed components.

This study could provide a valuable foundation for further research. Specifically, it would be worthwhile to investigate the impact of sintering, as explored by Ebrahimi et al. [8], on materials that contain metallic particles, like Filamet™ 6061 aluminum. This could offer insights into how post-processing techniques may enhance the thermal properties of FDM printed materials.

### 3.6. Potential Applications

Furthermore, this research indicates that high-thermal-conductivity composites hold promising potential for improving thermal insulation in construction, aerospace, and other industries. This can enhance energy efficiency, ultimately reducing both heating and cooling costs. In addition, these materials can be utilized in electronic devices, where temperature control is vital, to enhance heat dissipation, thus pointing towards another potential research direction. Custom 3D printing can benefit from materials with high thermal conductivity, allowing us to produce personalized components with specific thermal requirements. Future research could expand upon this study to include a wider array of polymers and metals for a more comprehensive understanding. Moreover, further research could explore the effects of post-treatments, like sintering and other methods, on improving the thermal conductivity of these materials, expanding knowledge in this field of study.

## 4. Conclusions

The rapid expansion and cost-effectiveness of additive manufacturing technologies, particularly fused deposition modeling (FDM), have significantly altered materials’ development for various applications, including the automotive industry. FDM enables the production of intricate parts with fewer processing steps, providing a versatile and economical solution for numerous industries. The thermal conductivity of materials used in additive manufacturing is critical for applications with thermal requirements. Several factors have been documented to influence thermal conductivity, including air presence within parts, material type, heat flow direction, and post-treatments that impact metal particles. Controlling thermal conductivity is crucial in optimizing the performance of these materials.

In this study, the thermal conductivity of seven materials with varying mechanical and thermal properties was analyzed. Highly precise measurements were obtained by employing a commercially available DTC-25 thermal conductivity measurement bench, adapted particularly for polymeric materials. The system’s stability, uncertainty, and calibration procedures were meticulously considered, ensuring the reliability and reproducibility of the results.

The results obtained allow us to conclude that the inclusion of metallic particles in thermoplastic materials leads to a significant improvement in their thermal conductivity. Our research findings demonstrate that materials like Filamet™ 6061 Aluminum, which contain over 65% metal and the rest PLA, exhibit high thermal conductivity, boasting a value of 0.40 ± 0.05 W/m·K. Compared to other materials, such as TPU, which is renowned for its mechanical and thermal resistance, with a thermal conductivity of 0.26 ± 0.05 W/m·K, Filamet™ 6061 Aluminum proves to be a more thermally efficient option. Consequently, the outcomes of this investigation highlight the potential benefits of integrating metallic inclusions to enhance thermal performance. It is important to note that the type and concentration of metallic particles have a substantial impact on thermal conductivity.

This research work aligns with previous studies, highlighting that elevated concentrations of metallic particles result in increased thermal conductivity. For instance, it has been demonstrated that copper particles with a concentration exceeding 20% result in a significant enhancement of thermal conductivity. It has been observed that reducing the air content within the material also enhances thermal conductivity. To minimize porosity, a sintering processes or post-processing can be executed, similar to the copper particle-containing materials studied.

Additionally, the inclusion of carbon fibers can further enhance thermal conductivity, although it is crucial to consider the fibers’ orientation and configuration. It has been determined that orientating fibers in the direction of heat flow leads to a noteworthy enhancement in thermal performance.

Finally, this research demonstrates the significance of thermal conductivity in additive manufacturing. It provides valuable insights into the impact of metal inclusions, particle types, and concentrations on thermal performance.

## Figures and Tables

**Table 1:** DTC-25 main characteristics [24].

Method	Guarded Heat Flow Meter
Standard Test Method	ASTM E1530
Sample Compatibility	Solids, pastes, liquids, thin films
Temperature Range	Near ambient
Thermal Conductivity Range	0.1 to 20 W/m·K
Thermal Resistance Range	0.0004 to 0.012 m^2^ K/W
Accuracy	±3%
Reproducibility	±2%

**Table 2:** Material key properties and applications.

Material	Key Properties	Key Applications
Aluminum (≈65%)	Excellent joining characteristics, good acceptance of applied coatings. Combines relatively high strength, good workability, and high resistance to corrosion. Widely available.	Aircraft fittings, camera lens mounts, electrical fittings and connectors, hinge pins, magneto parts, brake and hydraulic pistons, appliance fittings, valve parts.
PEEK	High-performance engineering thermoplastic that belongs to the family of polyketones. Exceptional mechanical, thermal, and chemical properties	Automotive, aerospace, medical and healthcare, electrical and electronic.
TPU	It has the characteristics of both plastic and rubber. Exhibits durability, excellent tensile strength, high elongation at break, and good load-bearing capacity.	Agriculture, automotive, seals and gaskets, textile coatings, sports and leisure, tubes and hoses.
ABS	Impact-resistant engineering thermoplastic made of three monomers: acrylonitrile, butadiene, and styrene. It is the preferred choice for structural applications due to its physical properties: high rigidity, resistance to impact, abrasion, and strain.	Automotive parts, electrical and electronic, household products, pipe fittings, sports and leisure.
PLA	Rapidly growing concerns related to environmental health and safety, limiting dependence on petrochemical raw materials, and reducing carbon footprint.	Food contact packaging, healthcare and medical industry, high-end structural applications, fiber and textile industries.
ULTEM	Combination of outstanding thermal (high temperature resistance, thermo-oxidative stability), mechanical (high strength-to-weight ratio), and electrical properties. ULTEM polyetherimide has found its place in high-performance applications.	Automotive, aerospace, electrical and electronic, metal replacement for industrial applications, disposable and re-usable medical applications.

**Table 3:** Material properties.

Material	Max Test Temp. (°C)
Aluminum (≈65%) [26]	55
PEEK [27]	140
TPU [28]	164
ABS [29]	81
PLA3080 [30]	55
ULTEM1010 [31]	213
ULTEM9085 [32]	153

**Table 4:** Study samples’ geometry.

Sample	Sample	Size	
		Thickness (mm)	Diameter (mm)
Aluminum (≈65%)	1	49.75	4.60
	2	50.00	9.55
PEEK	1	50.35	5.25
	2	49.90	10.00
TPU	1	50.35	5.05
	2	50.10	10.00
ABS	1	50.00	5.20
	2	50.00	10.10
PLA3080	1	50.40	5.00
	2	50.35	10.00
ULTEM1010	1	50.00	5.30
	2	50.00	10.30
ULTEM9085	1	50.00	5.10
	2	49.80	10.20

**Table 5:** Stability criterion. DTC-25.

Thickness (mm)	Stabilization Time (h)
5	3
10	3
15	4
20	5
25	6

**Table 6:** Thermal conductivity of each material.

Material	k (W/K·m)
Aluminum	0.40 ± 0.05
PEEK	0.25 ± 0.05
TPU	0.26 ± 0.05
ABS	0.22 ± 0.06
PLA3080	0.22 ± 0.06
ULTEM1010	0.20 ± 0.06
ULTEM9085	0.20 ± 0.06

## Data Availability

Data are contained within the article.
